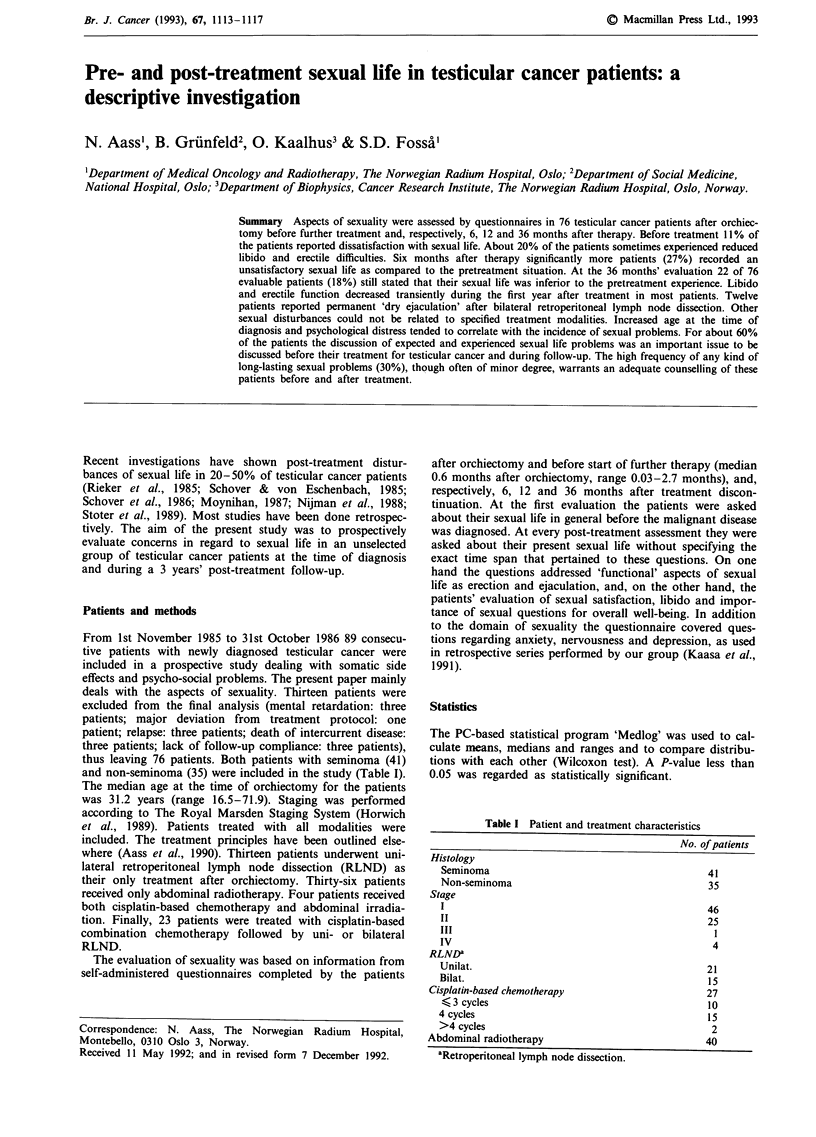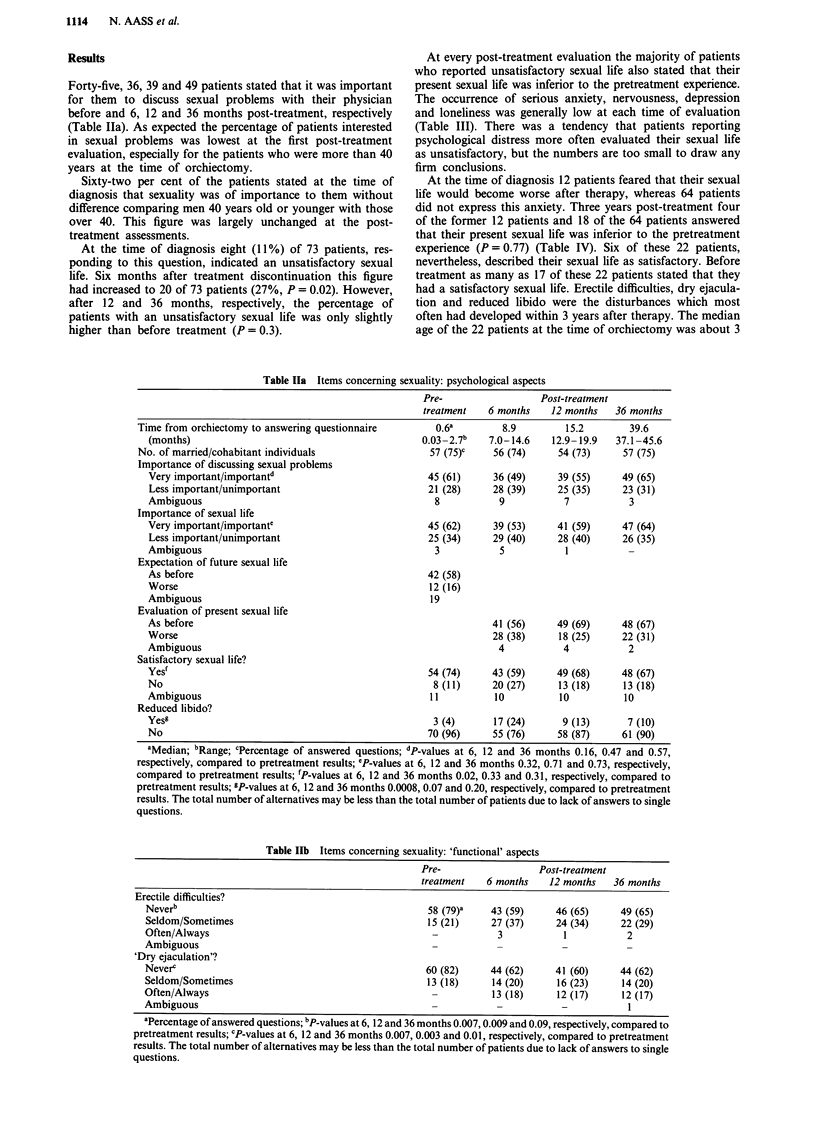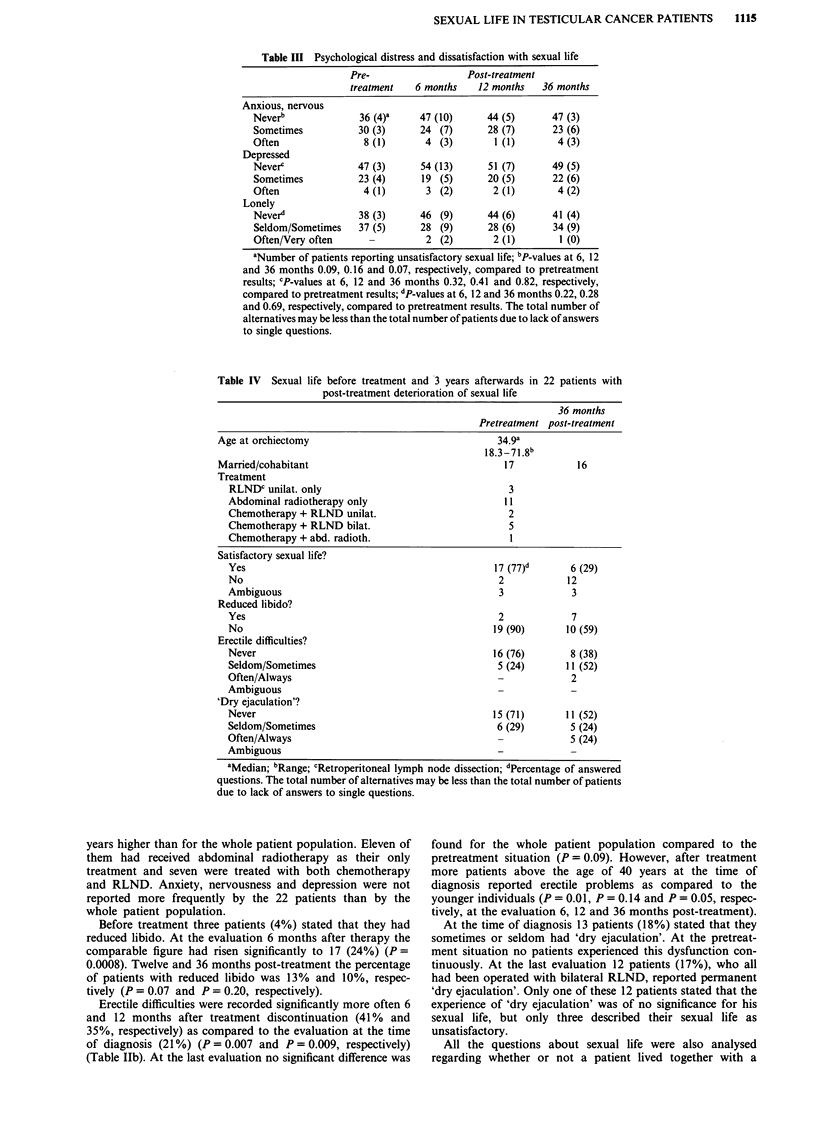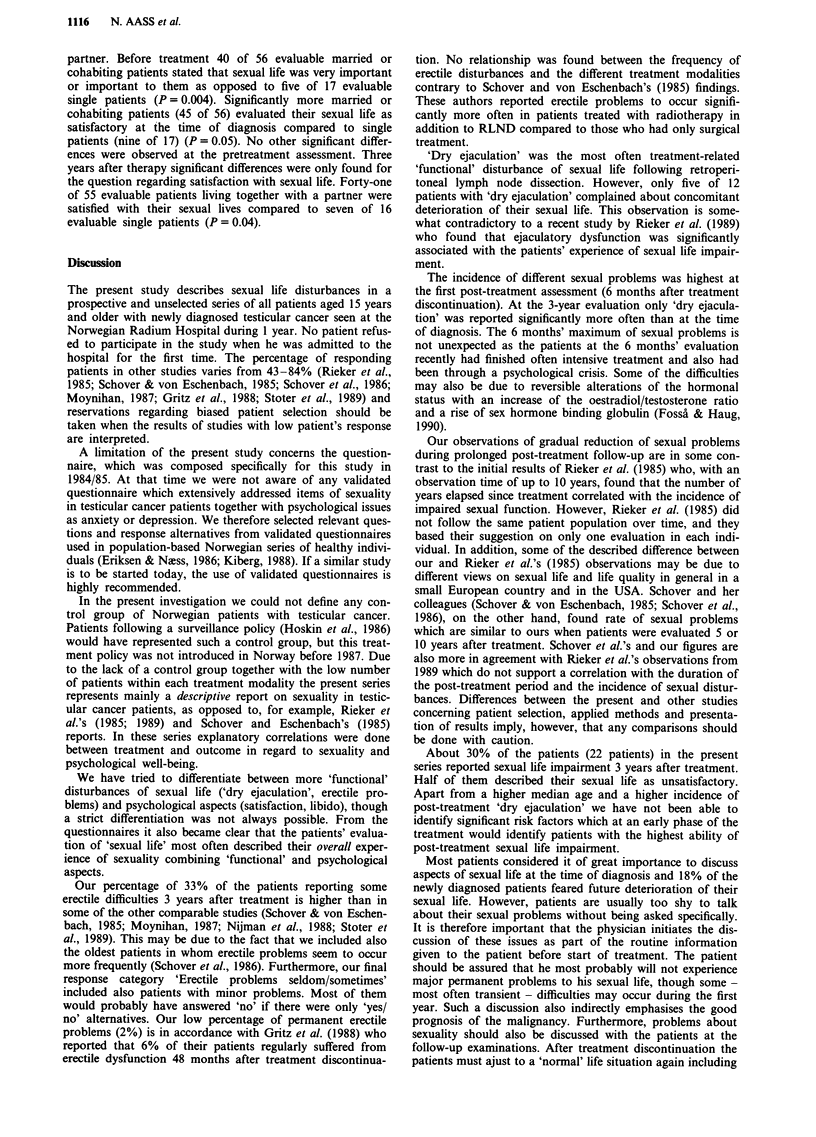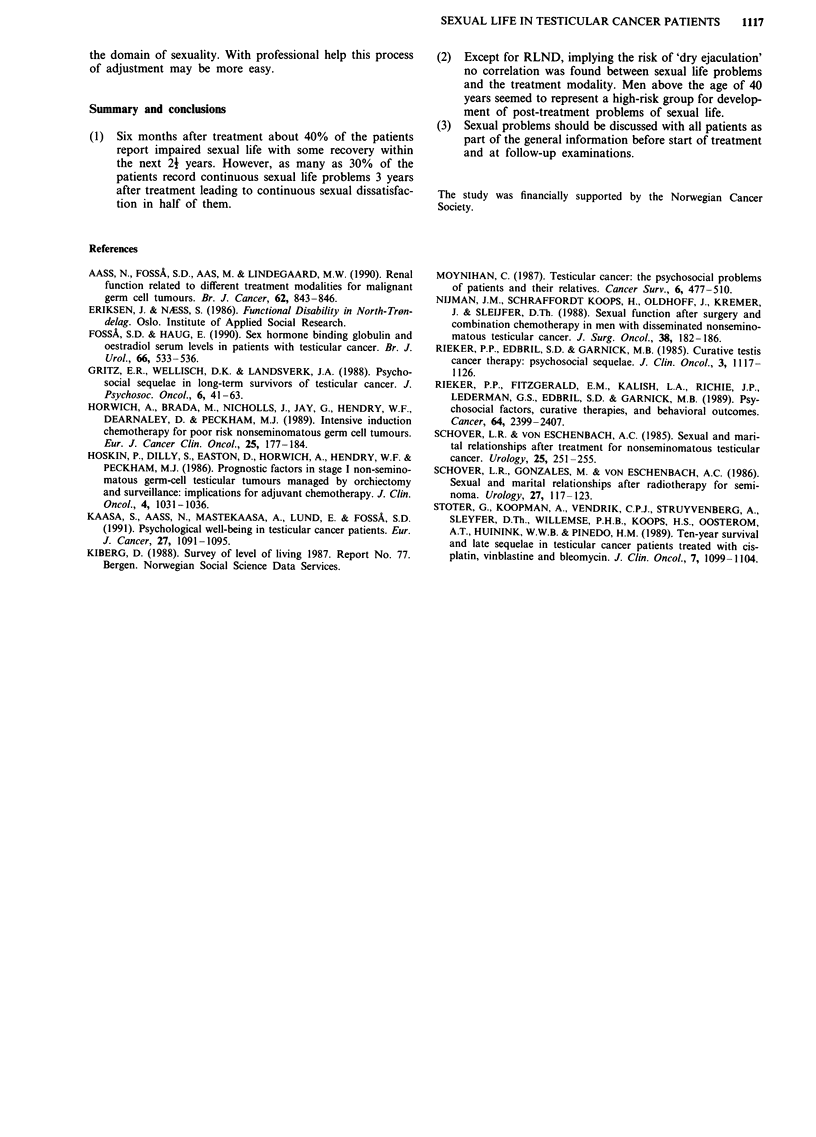# Pre- and post-treatment sexual life in testicular cancer patients: a descriptive investigation.

**DOI:** 10.1038/bjc.1993.204

**Published:** 1993-05

**Authors:** N. Aass, B. Grünfeld, O. Kaalhus, S. D. Fosså

**Affiliations:** Department of Medical Oncology and Radiotherapy, Norwegian Radium Hospital, Oslo.

## Abstract

Aspects of sexuality were assessed by questionnaires in 76 testicular cancer patients after orchiectomy before further treatment and, respectively, 6, 12 and 36 months after therapy. Before treatment 11% of the patients reported dissatisfaction with sexual life. About 20% of the patients sometimes experienced reduced libido and erectile difficulties. Six months after therapy significantly more patients (27%) recorded an unsatisfactory sexual life as compared to the pretreatment situation. At the 36 months' evaluation 22 of 76 evaluable patients (18%) still stated that their sexual life was inferior to the pretreatment experience. Libido and erectile function decreased transiently during the first year after treatment in most patients. Twelve patients reported permanent 'dry ejaculation' after bilateral retroperitoneal lymph node dissection. Other sexual disturbances could not be related to specified treatment modalities. Increased age at the time of diagnosis and psychological distress tended to correlate with the incidence of sexual problems. For about 60% of the patients the discussion of expected and experienced sexual life problems was an important issue to be discussed before their treatment for testicular cancer and during follow-up. The high frequency of any kind of long-lasting sexual problems (30%), though often of minor degree, warrants an adequate counselling of these patients before and after treatment.


Br. J. Cancer (1993), 67, 1113–1117 © Macmillan Press Ltd., 1993

### Pre- and post-treatment sexual life in testicular cancer patients: a descriptive investigation

N. Aass[1], B. Grünfeld[2], O. Kaalhus[3] & S.D. Fosså[1]

[1]*Department of Medical Oncology and Radiotherapy, The Norwegian Radium Hospital, Oslo;* [2]*Department of Social Medicine, National Hospital, Oslo;* [3]*Department of Biophysics, Cancer Research Institute, The Norwegian Radium Hospital, Oslo, Norway.*

**Summary** Aspects of sexuality were assessed by questionnaires in 76 testicular cancer patients after orchiectomy before further treatment and, respectively, 6, 12 and 36 months after therapy. Before treatment 11% of the patients reported dissatisfaction with sexual life. About 20% of the patients sometimes experienced reduced libido and erectile difficulties. Six months after therapy significantly more patients (27%) recorded an unsatisfactory sexual life as compared to the pretreatment situation. At the 36 months' evaluation 22 of 76 evaluable patients (18%) still stated that their sexual life was inferior to the pretreatment experience. Libido and erectile function decreased transiently during the first year after treatment in most patients. Twelve patients reported permanent 'dry ejaculation' after bilateral retroperitoneal lymph node dissection. Other sexual disturbances could not be related to specified treatment modalities. Increased age at the time of diagnosis and psychological distress tended to correlate with the incidence of sexual problems. For about 60% of the patients the discussion of expected and experienced sexual life problems was an important issue to be discussed before their treatment for testicular cancer and during follow-up. The high frequency of any kind of long-lasting sexual problems (30%), though often of minor degree, warrants an adequate counselling of these patients before and after treatment.

Recent investigations have shown post-treatment disturbances of sexual life in 20–50% of testicular cancer patients (Rieker *et al.*, 1985; Schover & von Eschenbach, 1985; Schover *et al.*, 1986; Moynihan, 1987; Nijman *et al.*, 1988; Stoter *et al.*, 1989). Most studies have been done retrospectively. The aim of the present study was to prospectively evaluate concerns in regard to sexual life in an unselected group of testicular cancer patients at the time of diagnosis and during a 3 years' post-treatment follow-up.

### Patients and methods

From 1st November 1985 to 31st October 1986 89 consecutive patients with newly diagnosed testicular cancer were included in a prospective study dealing with somatic side effects and psycho-social problems. The present paper mainly deals with the aspects of sexuality. Thirteen patients were excluded from the final analysis (mental retardation: three patients; major deviation from treatment protocol: one patient; relapse: three patients; death of intercurrent disease: three patients; lack of follow-up compliance: three patients), thus leaving 76 patients. Both patients with seminoma (41) and non-seminoma (35) were included in the study (Table I). The median age at the time of orchiectomy for the patients was 31.2 years (range 16.5–71.9). Staging was performed according to The Royal Marsden Staging System (Horwich *et al.*, 1989). Patients treated with all modalities were included. The treatment principles have been outlined elsewhere (Aass *et al.*, 1990). Thirteen patients underwent unilateral retroperitoneal lymph node dissection (RLND) as their only treatment after orchiectomy. Thirty-six patients received only abdominal radiotherapy. Four patients received both cisplatin-based chemotherapy and abdominal irradiation. Finally, 23 patients were treated with cisplatin-based combination chemotherapy followed by uni- or bilateral RLND.

The evaluation of sexuality was based on information from self-administered questionnaires completed by the patients after orchiectomy and before start of further therapy (median 0.6 months after orchiectomy, range 0.03–2.7 months), and, respectively, 6, 12 and 36 months after treatment discontinuation. At the first evaluation the patients were asked about their sexual life in general before the malignant disease was diagnosed. At every post-treatment assessment they were asked about their present sexual life without specifying the exact time span that pertained to these questions. On one hand the questions addressed 'functional' aspects of sexual life as erection and ejaculation, and, on the other hand, the patients' evaluation of sexual satisfaction, libido and importance of sexual questions for overall well-being. In addition to the domain of sexuality the questionnaire covered questions regarding anxiety, nervousness and depression, as used in retrospective series performed by our group (Kaasa *et al.*, 1991).

### Statistics

The PC-based statistical program 'Medlog' was used to calculate means, medians and ranges and to compare distributions with each other (Wilcoxon test). A *P*-value less than 0.05 was regarded as statistically significant.

**Table I** Patient and treatment characteristics

| | *No. of patients* |
|---|---|
| *Histology* | |
| Seminoma | 41 |
| Non-seminoma | 35 |
| *Stage* | |
| I | 46 |
| II | 25 |
| III | 1 |
| IV | 4 |
| *RLND*[a] | |
| Unilat. | 21 |
| Bilat. | 15 |
| *Cisplatin-based chemotherapy* | 27 |
| $\leqslant$3 cycles | 10 |
| 4 cycles | 15 |
| >4 cycles | 2 |
| Abdominal radiotherapy | 40 |

[a]Retroperitoneal lymph node dissection.

Correspondence: N. Aass, The Norwegian Radium Hospital, Montebello, 0310 Oslo 3, Norway.
Received 11 May 1992; and in revised form 7 December 1992.

### Results

Forty-five, 36, 39 and 49 patients stated that it was important for them to discuss sexual problems with their physician before and 6, 12 and 36 months post-treatment, respectively (Table IIa). As expected the percentage of patients interested in sexual problems was lowest at the first post-treatment evaluation, especially for the patients who were more than 40 years at the time of orchiectomy.

Sixty-two per cent of the patients stated at the time of diagnosis that sexuality was of importance to them without difference comparing men 40 years old or younger with those over 40. This figure was largely unchanged at the post-treatment assessments.

At the time of diagnosis eight (11%) of 73 patients, responding to this question, indicated an unsatisfactory sexual life. Six months after treatment discontinuation this figure had increased to 20 of 73 patients (27%, $P = 0.02$). However, after 12 and 36 months, respectively, the percentage of patients with an unsatisfactory sexual life was only slightly higher than before treatment ($P = 0.3$).

At every post-treatment evaluation the majority of patients who reported unsatisfactory sexual life also stated that their present sexual life was inferior to the pretreatment experience. The occurrence of serious anxiety, nervousness, depression and loneliness was generally low at each time of evaluation (Table III). There was a tendency that patients reporting psychological distress more often evaluated their sexual life as unsatisfactory, but the numbers are too small to draw any firm conclusions.

At the time of diagnosis 12 patients feared that their sexual life would become worse after therapy, whereas 64 patients did not express this anxiety. Three years post-treatment four of the former 12 patients and 18 of the 64 patients answered that their present sexual life was inferior to the pretreatment experience ($P = 0.77$) (Table IV). Six of these 22 patients, nevertheless, described their sexual life as satisfactory. Before treatment as many as 17 of these 22 patients stated that they had a satisfactory sexual life. Erectile difficulties, dry ejaculation and reduced libido were the disturbances which most often had developed within 3 years after therapy. The median age of the 22 patients at the time of orchiectomy was about 3

**Table IIa** Items concerning sexuality: psychological aspects

| | *Pre-treatment* | *Post-treatment* 6 months | 12 months | 36 months |
|---|---|---|---|---|
| Time from orchiectomy to answering questionnaire | 0.6[a] | 8.9 | 15.2 | 39.6 |
| (months) | 0.03–2.7[b] | 7.0–14.6 | 12.9–19.9 | 37.1–45.6 |
| No. of married/cohabitant individuals | 57 (75)[c] | 56 (74) | 54 (73) | 57 (75) |
| Importance of discussing sexual problems | | | | |
| Very important/important[d] | 45 (61) | 36 (49) | 39 (55) | 49 (65) |
| Less important/unimportant | 21 (28) | 28 (39) | 25 (35) | 23 (31) |
| Ambiguous | 8 | 9 | 7 | 3 |
| Importance of sexual life | | | | |
| Very important/important[e] | 45 (62) | 39 (53) | 41 (59) | 47 (64) |
| Less important/unimportant | 25 (34) | 29 (40) | 28 (40) | 26 (35) |
| Ambiguous | 3 | 5 | 1 | – |
| Expectation of future sexual life | | | | |
| As before | 42 (58) | | | |
| Worse | 12 (16) | | | |
| Ambiguous | 19 | | | |
| Evaluation of present sexual life | | | | |
| As before | | 41 (56) | 49 (69) | 48 (67) |
| Worse | | 28 (38) | 18 (25) | 22 (31) |
| Ambiguous | | 4 | 4 | 2 |
| Satisfactory sexual life? | | | | |
| Yes[f] | 54 (74) | 43 (59) | 49 (68) | 48 (67) |
| No | 8 (11) | 20 (27) | 13 (18) | 13 (18) |
| Ambiguous | 11 | 10 | 10 | 10 |
| Reduced libido? | | | | |
| Yes[g] | 3 (4) | 17 (24) | 9 (13) | 7 (10) |
| No | 70 (96) | 55 (76) | 58 (87) | 61 (90) |

[a]Median; [b]Range; [c]Percentage of answered questions; [d]*P*-values at 6, 12 and 36 months 0.16, 0.47 and 0.57, respectively, compared to pretreatment results; [e]*P*-values at 6, 12 and 36 months 0.32, 0.71 and 0.73, respectively, compared to pretreatment results; [f]*P*-values at 6, 12 and 36 months 0.02, 0.33 and 0.31, respectively, compared to pretreatment results; [g]*P*-values at 6, 12 and 36 months 0.0008, 0.07 and 0.20, respectively, compared to pretreatment results. The total number of alternatives may be less than the total number of patients due to lack of answers to single questions.

**Table IIb** Items concerning sexuality: 'functional' aspects

| | *Pre-treatment* | *Post-treatment* 6 months | 12 months | 36 months |
|---|---|---|---|---|
| Erectile difficulties? | | | | |
| Never[b] | 58 (79)[a] | 43 (59) | 46 (65) | 49 (65) |
| Seldom/Sometimes | 15 (21) | 27 (37) | 24 (34) | 22 (29) |
| Often/Always | – | 3 | 1 | 2 |
| Ambiguous | – | – | – | – |
| 'Dry ejaculation'? | | | | |
| Never[c] | 60 (82) | 44 (62) | 41 (60) | 44 (62) |
| Seldom/Sometimes | 13 (18) | 14 (20) | 16 (23) | 14 (20) |
| Often/Always | – | 13 (18) | 12 (17) | 12 (17) |
| Ambiguous | – | – | – | 1 |

[a]Percentage of answered questions; [b]*P*-values at 6, 12 and 36 months 0.007, 0.009 and 0.09, respectively, compared to pretreatment results; [c]*P*-values at 6, 12 and 36 months 0.007, 0.003 and 0.01, respectively, compared to pretreatment results. The total number of alternatives may be less than the total number of patients due to lack of answers to single questions.

**Table III** Psychological distress and dissatisfaction with sexual life

| | *Pre-treatment* | *Post-treatment 6 months* | *12 months* | *36 months* |
|---|---|---|---|---|
| Anxious, nervous | | | | |
| Never[b] | 36 (4)[a] | 47 (10) | 44 (5) | 47 (3) |
| Sometimes | 30 (3) | 24 (7) | 28 (7) | 23 (6) |
| Often | 8 (1) | 4 (3) | 1 (1) | 4 (3) |
| Depressed | | | | |
| Never[c] | 47 (3) | 54 (13) | 51 (7) | 49 (5) |
| Sometimes | 23 (4) | 19 (5) | 20 (5) | 22 (6) |
| Often | 4 (1) | 3 (2) | 2 (1) | 4 (2) |
| Lonely | | | | |
| Never[d] | 38 (3) | 46 (9) | 44 (6) | 41 (4) |
| Seldom/Sometimes | 37 (5) | 28 (9) | 28 (6) | 34 (9) |
| Often/Very often | – | 2 (2) | 2 (1) | 1 (0) |

[a]Number of patients reporting unsatisfactory sexual life; [b]*P*-values at 6, 12 and 36 months 0.09, 0.16 and 0.07, respectively, compared to pretreatment results; [c]*P*-values at 6, 12 and 36 months 0.32, 0.41 and 0.82, respectively, compared to pretreatment results; [d]*P*-values at 6, 12 and 36 months 0.22, 0.28 and 0.69, respectively, compared to pretreatment results. The total number of alternatives may be less than the total number of patients due to lack of answers to single questions.

**Table IV** Sexual life before treatment and 3 years afterwards in 22 patients with post-treatment deterioration of sexual life

| | *Pretreatment* | *36 months post-treatment* |
|---|---|---|
| Age at orchiectomy | 34.9[a] 18.3–71.8[b] | |
| Married/cohabitant | 17 | 16 |
| Treatment | | |
| RLND[c] unilat. only | 3 | |
| Abdominal radiotherapy only | 11 | |
| Chemotherapy + RLND unilat. | 2 | |
| Chemotherapy + RLND bilat. | 5 | |
| Chemotherapy + abd. radioth. | 1 | |
| Satisfactory sexual life? | | |
| Yes | 17 (77)[d] | 6 (29) |
| No | 2 | 12 |
| Ambiguous | 3 | 3 |
| Reduced libido? | | |
| Yes | 2 | 7 |
| No | 19 (90) | 10 (59) |
| Erectile difficulties? | | |
| Never | 16 (76) | 8 (38) |
| Seldom/Sometimes | 5 (24) | 11 (52) |
| Often/Always | – | 2 |
| Ambiguous | – | – |
| 'Dry ejaculation'? | | |
| Never | 15 (71) | 11 (52) |
| Seldom/Sometimes | 6 (29) | 5 (24) |
| Often/Always | – | 5 (24) |
| Ambiguous | – | – |

[a]Median; [b]Range; [c]Retroperitoneal lymph node dissection; [d]Percentage of answered questions. The total number of alternatives may be less than the total number of patients due to lack of answers to single questions.

years higher than for the whole patient population. Eleven of them had received abdominal radiotherapy as their only treatment and seven were treated with both chemotherapy and RLND. Anxiety, nervousness and depression were not reported more frequently by the 22 patients than by the whole patient population.

Before treatment three patients (4%) stated that they had reduced libido. At the evaluation 6 months after therapy the comparable figure had risen significantly to 17 (24%) ($P = 0.0008$). Twelve and 36 months post-treatment the percentage of patients with reduced libido was 13% and 10%, respectively ($P = 0.07$ and $P = 0.20$, respectively).

Erectile difficulties were recorded significantly more often 6 and 12 months after treatment discontinuation (41% and 35%, respectively) as compared to the evaluation at the time of diagnosis (21%) ($P = 0.007$ and $P = 0.009$, respectively) (Table IIb). At the last evaluation no significant difference was found for the whole patient population compared to the pretreatment situation ($P = 0.09$). However, after treatment more patients above the age of 40 years at the time of diagnosis reported erectile problems as compared to the younger individuals ($P = 0.01$, $P = 0.14$ and $P = 0.05$, respectively, at the evaluation 6, 12 and 36 months post-treatment).

At the time of diagnosis 13 patients (18%) stated that they sometimes or seldom had 'dry ejaculation'. At the pretreatment situation no patients experienced this dysfunction continuously. At the last evaluation 12 patients (17%), who all had been operated with bilateral RLND, reported permanent 'dry ejaculation'. Only one of these 12 patients stated that the experience of 'dry ejaculation' was of no significance for his sexual life, but only three described their sexual life as unsatisfactory.

All the questions about sexual life were also analysed regarding whether or not a patient lived together with a partner. Before treatment 40 of 56 evaluable married or cohabiting patients stated that sexual life was very important or important to them as opposed to five of 17 evaluable single patients ($P = 0.004$). Significantly more married or cohabiting patients (45 of 56) evaluated their sexual life as satisfactory at the time of diagnosis compared to single patients (nine of 17) ($P = 0.05$). No other significant differences were observed at the pretreatment assessment. Three years after therapy significant differences were only found for the question regarding satisfaction with sexual life. Forty-one of 55 evaluable patients living together with a partner were satisfied with their sexual lives compared to seven of 16 evaluable single patients ($P = 0.04$).

### Discussion

The present study describes sexual life disturbances in a prospective and unselected series of all patients aged 15 years and older with newly diagnosed testicular cancer seen at the Norwegian Radium Hospital during 1 year. No patient refused to participate in the study when he was admitted to the hospital for the first time. The percentage of responding patients in other studies varies from 43–84% (Rieker et al., 1985; Schover & von Eschenbach, 1985; Schover et al., 1986; Moynihan, 1987; Gritz et al., 1988; Stoter et al., 1989) and reservations regarding biased patient selection should be taken when the results of studies with low patient's response are interpreted.

A limitation of the present study concerns the questionnaire, which was composed specifically for this study in 1984/85. At that time we were not aware of any validated questionnaire which extensively addressed items of sexuality in testicular cancer patients together with psychological issues as anxiety or depression. We therefore selected relevant questions and response alternatives from validated questionnaires used in population-based Norwegian series of healthy individuals (Eriksen & Næss, 1986; Kiberg, 1988). If a similar study is to be started today, the use of validated questionnaires is highly recommended.

In the present investigation we could not define any control group of Norwegian patients with testicular cancer. Patients following a surveillance policy (Hoskin et al., 1986) would have represented such a control group, but this treatment policy was not introduced in Norway before 1987. Due to the lack of a control group together with the low number of patients within each treatment modality the present series represents mainly a *descriptive* report on sexuality in testicular cancer patients, as opposed to, for example, Rieker et al.'s (1985; 1989) and Schover and Eschenbach's (1985) reports. In these series explanatory correlations were done between treatment and outcome in regard to sexuality and psychological well-being.

We have tried to differentiate between more 'functional' disturbances of sexual life ('dry ejaculation', erectile problems) and psychological aspects (satisfaction, libido), though a strict differentiation was not always possible. From the questionnaires it also became clear that the patients' evaluation of 'sexual life' most often described their *overall* experience of sexuality combining 'functional' and psychological aspects.

Our percentage of 33% of the patients reporting some erectile difficulties 3 years after treatment is higher than in some of the other comparable studies (Schover & von Eschenbach, 1985; Moynihan, 1987; Nijman et al., 1988; Stoter et al., 1989). This may be due to the fact that we included also the oldest patients in whom erectile problems seem to occur more frequently (Schover et al., 1986). Furthermore, our final response category 'Erectile problems seldom/sometimes' included also patients with minor problems. Most of them would probably have answered 'no' if there were only 'yes/no' alternatives. Our low percentage of permanent erectile problems (2%) is in accordance with Gritz et al. (1988) who reported that 6% of their patients regularly suffered from erectile dysfunction 48 months after treatment discontinuation. No relationship was found between the frequency of erectile disturbances and the different treatment modalities contrary to Schover and von Eschenbach's (1985) findings. These authors reported erectile problems to occur significantly more often in patients treated with radiotherapy in addition to RLND compared to those who had only surgical treatment.

'Dry ejaculation' was the most often treatment-related 'functional' disturbance of sexual life following retroperitoneal lymph node dissection. However, only five of 12 patients with 'dry ejaculation' complained about concomitant deterioration of their sexual life. This observation is somewhat contradictory to a recent study by Rieker et al. (1989) who found that ejaculatory dysfunction was significantly associated with the patients' experience of sexual life impairment.

The incidence of different sexual problems was highest at the first post-treatment assessment (6 months after treatment discontinuation). At the 3-year evaluation only 'dry ejaculation' was reported significantly more often than at the time of diagnosis. The 6 months' maximum of sexual problems is not unexpected as the patients at the 6 months' evaluation recently had finished often intensive treatment and also had been through a psychological crisis. Some of the difficulties may also be due to reversible alterations of the hormonal status with an increase of the oestradiol/testosterone ratio and a rise of sex hormone binding globulin (Fosså & Haug, 1990).

Our observations of gradual reduction of sexual problems during prolonged post-treatment follow-up are in some contrast to the initial results of Rieker et al. (1985) who, with an observation time of up to 10 years, found that the number of years elapsed since treatment correlated with the incidence of impaired sexual function. However, Rieker et al. (1985) did not follow the same patient population over time, and they based their suggestion on only one evaluation in each individual. In addition, some of the described difference between our and Rieker et al.'s (1985) observations may be due to different views on sexual life and life quality in general in a small European country and in the USA. Schover and her colleagues (Schover & von Eschenbach, 1985; Schover et al., 1986), on the other hand, found rate of sexual problems which are similar to ours when patients were evaluated 5 or 10 years after treatment. Schover et al.'s and our figures are also more in agreement with Rieker et al.'s observations from 1989 which do not support a correlation with the duration of the post-treatment period and the incidence of sexual disturbances. Differences between the present and other studies concerning patient selection, applied methods and presentation of results imply, however, that any comparisons should be done with caution.

About 30% of the patients (22 patients) in the present series reported sexual life impairment 3 years after treatment. Half of them described their sexual life as unsatisfactory. Apart from a higher median age and a higher incidence of post-treatment 'dry ejaculation' we have not been able to identify significant risk factors which at an early phase of the treatment would identify patients with the highest ability of post-treatment sexual life impairment.

Most patients considered it of great importance to discuss aspects of sexual life at the time of diagnosis and 18% of the newly diagnosed patients feared future deterioration of their sexual life. However, patients are usually too shy to talk about their sexual problems without being asked specifically. It is therefore important that the physician initiates the discussion of these issues as part of the routine information given to the patient before start of treatment. The patient should be assured that he most probably will not experience major permanent problems to his sexual life, though some – most often transient – difficulties may occur during the first year. Such a discussion also indirectly emphasises the good prognosis of the malignancy. Furthermore, problems about sexuality should also be discussed with the patients at the follow-up examinations. After treatment discontinuation the patients must ajust to a 'normal' life situation again including the domain of sexuality. With professional help this process of adjustment may be more easy.

### Summary and conclusions

(1) Six months after treatment about 40% of the patients report impaired sexual life with some recovery within the next 2½ years. However, as many as 30% of the patients record continuous sexual life problems 3 years after treatment leading to continuous sexual dissatisfaction in half of them.

(2) Except for RLND, implying the risk of 'dry ejaculation' no correlation was found between sexual life problems and the treatment modality. Men above the age of 40 years seemed to represent a high-risk group for development of post-treatment problems of sexual life.

(3) Sexual problems should be discussed with all patients as part of the general information before start of treatment and at follow-up examinations.

The study was financially supported by the Norwegian Cancer Society.

### References

AASS, N., FOSSÅ, S.D., AAS, M. & LINDEGAARD, M.W. (1990). Renal function related to different treatment modalities for malignant germ cell tumours. *Br. J. Cancer*, **62**, 843–846.

ERIKSEN, J. & NÆSS, S. (1986). *Functional Disability in North-Trøndelag*. Oslo. Institute of Applied Social Research.

FOSSÅ, S.D. & HAUG, E. (1990). Sex hormone binding globulin and oestradiol serum levels in patients with testicular cancer. *Br. J. Urol.*, **66**, 533–536.

GRITZ, E.R., WELLISCH, D.K. & LANDSVERK, J.A. (1988). Psychosocial sequelae in long-term survivors of testicular cancer. *J. Psychosoc. Oncol.*, **6**, 41–63.

HORWICH, A., BRADA, M., NICHOLLS, J., JAY, G., HENDRY, W.F., DEARNALEY, D. & PECKHAM, M.J. (1989). Intensive induction chemotherapy for poor risk nonseminomatous germ cell tumours. *Eur. J. Cancer Clin. Oncol.*, **25**, 177–184.

HOSKIN, P., DILLY, S., EASTON, D., HORWICH, A., HENDRY, W.F. & PECKHAM, M.J. (1986). Prognostic factors in stage I non-seminomatous germ-cell testicular tumours managed by orchiectomy and surveillance: implications for adjuvant chemotherapy. *J. Clin. Oncol.*, **4**, 1031–1036.

KAASA, S., AASS, N., MASTEKAASA, A., LUND, E. & FOSSÅ, S.D. (1991). Psychological well-being in testicular cancer patients. *Eur. J. Cancer*, **27**, 1091–1095.

KIBERG, D. (1988). Survey of level of living 1987. Report No. 77. Bergen. Norwegian Social Science Data Services.

MOYNIHAN, C. (1987). Testicular cancer: the psychosocial problems of patients and their relatives. *Cancer Surv.*, **6**, 477–510.

NIJMAN, J.M., SCHRAFFORDT KOOPS, H., OLDHOFF, J., KREMER, J. & SLEIJFER, D.Th. (1988). Sexual function after surgery and combination chemotherapy in men with disseminated nonseminomatous testicular cancer. *J. Surg. Oncol.*, **38**, 182–186.

RIEKER, P.P., EDBRIL, S.D. & GARNICK, M.B. (1985). Curative testis cancer therapy: psychosocial sequelae. *J. Clin. Oncol.*, **3**, 1117–1126.

RIEKER, P.P., FITZGERALD, E.M., KALISH, L.A., RICHIE, J.P., LEDERMAN, G.S., EDBRIL, S.D. & GARNICK, M.B. (1989). Psychosocial factors, curative therapies, and behavioral outcomes. *Cancer*, **64**, 2399–2407.

SCHOVER, L.R. & VON ESCHENBACH, A.C. (1985). Sexual and marital relationships after treatment for nonseminomatous testicular cancer. *Urology*, **25**, 251–255.

SCHOVER, L.R., GONZALES, M. & VON ESCHENBACH, A.C. (1986). Sexual and marital relationships after radiotherapy for seminoma. *Urology*, **27**, 117–123.

STOTER, G., KOOPMAN, A., VENDRIK, C.P.J., STRUYVENBERG, A., SLEYFER, D.Th., WILLEMSE, P.H.B., KOOPS, H.S., OOSTEROM, A.T., HUININK, W.W.B. & PINEDO, H.M. (1989). Ten-year survival and late sequelae in testicular cancer patients treated with cisplatin, vinblastine and bleomycin. *J. Clin. Oncol.*, **7**, 1099–1104.